# COVID-19 and its impact on healthcare services provided to patients with COPD: a qualitative study

**DOI:** 10.1136/bmjresp-2025-003196

**Published:** 2025-12-25

**Authors:** Abdullah Aljahan, Rachel E Jordan, James Hodgkinson

**Affiliations:** 1 College of Pharmacy, Prince Sattam bin Abdulaziz University, Al Kharj, Saudi Arabia; 2 College of Medicine and Health, University of Birmingham, Birmingham, UK

**Keywords:** COVID-19, Pulmonary Disease, Chronic Obstructive, Telemedicine

## Abstract

**Introduction:**

The COVID-19 pandemic significantly impacted primary healthcare for chronic disease patients, including those with chronic obstructive pulmonary disease (COPD). Information about whether virtual or remote consultations were effective or acceptable to patients with COPD is sparse. E-consultation and telephone triage strategies were deployed, with the vast majority of triaged consultations taking place over the phone or through video calls. This study explored the views of patients with COPD and providers on the effects of the pandemic on their health and healthcare, and barriers to/enablers of remote consultations.

**Methods:**

12 semi-structured interviews with open-ended questions were conducted via telephone/Zoom among patients with COPD and healthcare professionals (HCPs) in the West Midlands, UK. Patients with COPD were recruited from the Birmingham Lung Improvement StudieS (BLISS) cohort. HCPs were approached via an advert distributed through general practitioner practices that were part of the BLISS cohort; brief adverts were disseminated to several professional organisations throughout the West Midlands area and social media. Interviews were analysed using Braun and Clarke’s six steps of thematic analysis.

**Results:**

Three major themes around coping and life adjustment, NHS service accessibility and healthcare inequalities related to e-health literacy.

**Conclusion:**

Post-pandemic priorities should include enhancing remote consultation training for providers, with a focus on rapport-building and care delivery. Equally important are providing flexible healthcare access, continuity of care and targeted support for vulnerable patients with COPD to maintain essential services during crises.

**WHAT IS ALREADY KNOWN ON THIS TOPIC**

- Remote consultations expanded rapidly during COVID-19, but evidence on their acceptability and impact for chronic obstructive pulmonary disease (COPD) care has been limited.

**WHAT THIS STUDY ADDS**

- Interviews with patients with COPD and healthcare professionals (HCPs) in the West Midlands identified three core themes—coping and life adjustment, NHS service accessibility and e-health literacy inequalities—that shaped barriers and enablers to remote consultations. Older adults with more severe COPD preferred face-to-face consultations citing barriers related to access and use of technology. HCPs acknowledged the suitability of remote consultations for the stable patient, but not where there was clinical uncertainty.

**HOW THIS STUDY MIGHT AFFECT RESEARCH, PRACTICE OR POLICY**

- Findings support post-pandemic priorities of training clinicians in rapport building for remote care, enabling flexible and continuous access to care, and targeting support for vulnerable patients, while exercising caution about deprivation-related barriers and evaluating cost effectiveness before wider rollout

## Introduction

 Chronic obstructive pulmonary disease (COPD) is a progressive lung condition which imposes a considerable medical and financial burden on individuals, the healthcare system and society. Worldwide, approximately 328 million individuals are diagnosed with COPD.^1^ Individuals with COPD experience increasing risk of exacerbations as their disease progresses, usually triggered by respiratory viral infections,^2^ which are one of the most common causes of hospital admissions.

During the COVID-19 pandemic, a COPD diagnosis increased risks of hospitalisation and mortality^3^ by approximately 3.75 fold.^4^,^6^ To lessen the likelihood of spreading COVID-19 among patients and healthcare professionals in the UK, the NHS began offering remote consultations. E-consultation and telephone triage strategies were deployed,^7^ with the vast majority of triaged consultations taking place over the phone or through video calls during the pandemic.^8^

There is some evidence that patients with COPD were particularly negatively affected in terms of loneliness and isolation due to lockdown,^9^ inability to exercise and loss of function,^9^,^11^ the challenges of using mobile and electronic devices for communication^12 13^ and changes in access to healthcare, which required the use of such technology.^14^ Conversely, patients with COPD may have had better access to other members of the healthcare team such as pharmacists than previously.^9^ A recent UK study^15^ employed discrete event simulation to construct a tool evaluating COVID-19 restriction impacts on COPD management. Comparing four pandemic scenarios over a year (no, mild, stricter, severe restrictions), the study revealed a trade-off: stricter restrictions reduced COPD admissions but potentially diminished clinic visit quality.

A virtual consultation methodology has yet to be shown to be successful or popular.^16 17^ The COVID-19 pandemic acted as a catalyst for the rapid advancement of telemedicine and remote care models, enabling patients to access healthcare services from home. This qualitative study aimed to explore the impact of the pandemic on the healthcare of patients with COPD from the perspective of both patients and healthcare professionals, aiming to understand their experiences of remote consultations during the pandemic and identify any aspects which might be useful post-pandemic.

## Methods

### Study design

Qualitative study conducted among patients with COPD and healthcare professionals in the West Midlands, UK, from April to August 2021. Semi-structured interviews with open-ended questions were conducted by a solo researcher (AA) using telephone or Zoom.

### Patient and public involvement

An advisory group composed of pharmacy champions, clinical and community pharmacists, a nurse specialising in COPD, expert researchers in COPD and two patients diagnosed with COPD all reviewed the interview topic guide and study materials, and their feedback helped to revise the content, wording and format. Three patients diagnosed with COPD were asked to pilot the interview to ensure clarity and relevance, and their feedback was incorporated before data collection.

### Recruitment

Patients with COPD were purposively sampled from The Birmingham Lung Improvement StudieS (BLISS) COPD cohort.^18^ The invitation letter, Patient Information Sheet (PIS), reply slip and consent form were sent to all potentially eligible participants, with reminders to non-responders after 3 weeks and 6 weeks.

Healthcare professionals (HCPs) (general practitioners (GPs), nurses and in-house pharmacists) were purposively sampled and recruited through two separate activities. Invitations (with PIS, reply slip and consent form) were emailed to GP practices that took part in the BLISS cohort with reminders to non-responders after 3 weeks and 6 weeks. A brief advert about the study was also disseminated via the Clinical Research Network newsletter (Participate), the Primary Care Respiratory Society newsletter (In Touch), the Local Pharmacy Committee in West Midlands and social media. The advert briefly described the study and provided an email address for anyone interested in finding out more. Those individuals were then sent the invitation letter, Participants Information Sheet, reply slip and consent form via email.

### Interview process and content

Interviews were conducted via telephone or through Zoom according to participants’ preference and recorded with the participants’ consent. Safeguards included capacity checks, plain language consent, pause/withdraw rights and a brief distress protocol with clinical signposting if needed. Three pilot interviews with patients with COPD were conducted with members of the patient advisory group and two pilot interviews with HCPs to determine the clarity of the interview questions. After conducting the pilot interviews, participants found the questions clear and appropriate, so we did not make any changes to the interview guides. Interview topic guides were developed using the Theoretical Domains Framework.^19^ The topic guide (online supplemental appendix 1) for patients with COPD comprised questions related to their views and experiences of the accessibility of healthcare services and utilisation of remote consultations, the impact of COVID-19 and pandemic service changes on the management of their COPD and their inhaler use. The topic guide for HCPs covered similar questions from the point of view of the HCP.

Recruitment of participants continued until data saturation was achieved.^20^ Data saturation was achieved concurrently with coding and iterative reviewing during data collection, with recruitment stopped if three consecutive interviews did not produce new themes, suggesting there was enough richness in the data’s ability to address the research questions.^20^ Member checking was not performed for practical reasons to minimise participant burden and repeat contact. Transcripts and preliminary findings were not returned to participants for the same reasons. However, credibility was strengthened through independent double-coding of (n/N, eg, 6/12, 50%) transcripts with consensus (senior adjudication if needed), regular team debriefs and a maintained audit trail.

### Researcher characteristics and reflexivity

Prior to the fieldwork, the lead researcher completed a 2-month university module in qualitative methods, with formal instruction and supervised practice in semistructured interviewing. AA was a pharmacist and medication therapy management professional with limited knowledge of the UK context. This gave him insight into non-adherence and pharmacists’ problems, but less into local context. As a countermeasure, he started the interviews by defining himself as a PhD researcher and not a pharmacist, so the interviews would be informal, and he attempted to remain neutral while interviewing and analysing. He took steps to minimise bias by acknowledging his experience as a pharmacist and challenging his assumptions in doing so.

### Data collection details

Eight patients with COPD were interviewed (five men, three women), age range was 62–90 years old, severity of disease based on the four traditional Global Initiative for Chronic Obstructive Lung Disease grades of severity of airflow obstruction in COPD.^21^ The participants ranged from moderate to very severe COPD. Four HCPs were interviewed (all GPs) with age range 30–50 years old. 75% of interviewees had four or more years of practice experience (table 1). The Index of Multiple Deprivation ^22^ ranks small areas by combining data on income, employment, health, education, crime, housing and environment. It groups areas into ten levels, with one being the most deprived and 10 the least, showing how areas compare in terms of disadvantage. We collected sociodemographic data like sex, age, ethnicity, education and deprivation decile based on patient address and self-report, along with clinical details on physical activity, disease severity, diagnosis length and treatment adherence from questionnaires and medical records. Field notes were not kept; context and reflexive insights were captured in post-interview memos and used in analysis.

**Table 1:** Sociodemographic and other characteristics of the participants

	Patients (n=8)	Healthcare professionals (n=4)
Sex		
Men	5 (62.5%)	2 (0%)
Women	3 (37.5%)	2 (0%)
Not declared	0 (0%)	0 (0%)
Average age (years)		
Mean (SD)	78.5 (7.4)	20–30 0 (0%) 31–40 2 (50%) 41–50 2 (50%) 51–60 0 (0%) 61–70 (0%)
Ethnicity		
White British	7 (87.5%)	1 (25%)
White Irish	1 (12.5%)	0 (0%)
British Indian	0 (0%)	2 (50%)
Other	0 (0%)	1 (25%)
Education		
Elementary	0 (0%)	–
Middle school	0 (0%)	–
High school	3 (37.5%)	–
Diploma	2 (25%)	–
Bachelor	0 (0%)	3 (75%)
Master	0 (0%)	1 (0%)
PhD	0 (0%)	0 (0%)
None	3 (37.5%)	–
Index of Multiple Deprivation Decile		
1	1 (12.5%)	2 (50%)
2	2 (25%)	0 (0%)
3	0 (0%)	0 (0%)
4	1 (12.5%)	1 (25%)
5	2 (25%)	0 (0%)
6	1 (12.5%)	0 (0%)
7	0 (0%)	0 (0%)
8	0 (0%)	0 (0%)
9	1 (12.5%)	1 (25%)
Length of diagnosis (years)		
1–4	0 (0%)	–
5–10	3 (37.5%)	–
11–14	2 (25%)	–
15–19	0 (0%)	–
≥20	3 (37.5)	–
COPD severity		
Mild	0 (0%)	–
Moderate	1 (12.5%)	–
Severe	6 (75%)	–
Very severe	1 (12.5%)	–
Specialisation		
General practitioners	–	4 (100%)
Nurse	–	0 (0%)
Pharmacists	–	0 (0%)
Length of work experience (years)		
1	–	0 (0%)
2	–	1 (25%)
3	–	0 (0%)
4	–	1 (25%)
5	–	2 (50%)

COPD, chronic obstructive pulmonary disease.

### Data analysis

The lead researcher (AA) transcribed all interviews verbatim. The set of criteria for judging qualitative research developed by Lincoln and Guba,^23^ who suggested that credibility, transferability, dependability and confirmability be considered, was followed. In addition, the concept of reflexivity was considered by looking at the researcher’s background/ position to consider how those might influence the research process.^24 25^

An inductive approach^26^ was taken to identify themes using the Braun and Clarke^27^ six-step approach. Data analysis started during the early stages of data collection following each interview, enabling immediate engagement in the data set and the identification and incorporation of any new themes for subsequent interviews. Alongside the lead analyst, two co-authors checked transcripts against the audio recording and collaboratively refined the codebook, double-coding a subset and resolving any differences through discussion with all the authors. A summary of the coding tree with themes and subthemes is provided in the online supplemental data. The data were imported into qualitative analysis package NVivo software.^28^

## Results

536 participants were directly invited to take part in this study (243 by email, 293 by letter). A wider audience was invited via social media, although this is not quantifiable. 12 participants were recruited. Recorded interview length was between 21 and 60 min with an average of 39 min.

### Summary of themes

We identified three major themes within the data: (a) coping and life adjustment, (b) the accessibility of the NHS services and (c) health inequalities and e-health literacy. Each major theme had several subthemes (table 2).

**Table 2:** Major themes and subthemes by participant group

Themes	Subthemes and percentages of appearance	Patients (n=8)	Healthcare professionals (n=4)
Coping and life adjustment	Changes to lifestyle (37.5% of patients)	x	
	Impact of isolation, shielding, masking (62.5% of patients)	x	
	Self-management (carer dependency and caring roles) (37.5% of patients)	x	
Accessibility of the NHS services	Limited access to health facility (HCP appointments, follow-up) (75% of patients and 100% of healthcare professionals)	x	x
	Frustrations about the services provided by NHS (37.5% of patients)	x	
	Beliefs about NHS capacity (37.5% of patients)	x	
Health inequalities and e-health literacy	Patients’ digital disadvantage (100% of both patients and healthcare professionals)	x	x
	Inhaler technique (100% of healthcare professionals)		x

HCP, healthcare professionals.

### Remote consultations: overview of findings

Most participants preferred face-to-face care, especially older adults and those with more severe COPD, citing low digital confidence and communication challenges; a minority welcomed remote options during the pandemic for safety and convenience. Clinicians found remote care workable for stable patients and routine reviews, but were cautious for suspected exacerbations, diagnosis and where non-verbal cues or objective assessment were needed.

Among patients, common barriers included limited digital access and skills, dislike of video platforms, hearing or wider communication difficulties, a preference for in-person examination and greater challenges for those living in more deprived areas. Offsetting these, key enablers were perceived infection safety during the pandemic, convenience for routine issues and a greater willingness to use telephone over video when remote contact was needed.

From clinicians’ and service perspectives, barriers centred on diagnostic uncertainty without physical examination or non-verbal cues, safety concerns when managing potential respiratory exacerbations remotely and difficulty quantifying change in symptoms at a distance. Enablers included the use of triage and structured templates (eg, collecting COPD Assessment Test (CAT) scores before appointments), flexible scheduling, the ability in some settings to spend more time on calls and routinely offering a face-to-face option on request.

### Coping and life adjustment

#### Changes to lifestyle

Patients with COPD stated that lifestyle restriction was inevitable when the government imposed it.

The problem at the moment, we've not been seeing anybody because of the COVID…My daughter and myself, we’ve been both in very strict isolation. So I don't really get out too much, I mean at all at the moment. (Patient 1)

Well, I was in Scotland for the best part of a year with my partner, and we obviously couldn't go anywhere. We were in isolation then, but that is only because it was forced upon us by the government (Patient 8)

#### Physical activity

Two patients talked about physical activity before, during and after the pandemic, especially the difficulty in regaining physical fitness after restrictions were lifted.

No I am not able to walk now, and since we've had this pandemic, I am not going out at all. It made my walking worse (Patient 2)

#### Impact of isolation, shielding and masking

Aside from physical impacts, the pandemic of COVID-19 possibly led to increased mental ill-health among patients with COPD as a result of advised preventative practices such as social isolation and quarantine.

Well, I haven't been out at all. I don't mix with other people. In the last two years I have not been able to go out. So I miss all the local areas like (town in the West Midlands) and others (Patient 5)

I’m shielding, I don’t mix with other people (Patient 6)

#### Self-management (carer dependency and caring roles)

A patient with COPD with serious health conditions perceived that taking care of a family member during the pandemic was challenging.

It is affecting me with the COVID quite a bit. Plus my daughter’s a very sick person so as to care for her as well… In the past couple of months, I've had a problem with my spine, problem with my hip, and also I've had pneumonia. (Patient 1)

Three patients with COPD were aware that caregivers such as family members and close friends and neighbours had to help them more than usual.

Because of COVID my daughter has been picking up my prescriptions, and my next-door neighbour’s been picking up my prescriptions, and sometimes the pharmacy itself (name masked) Pharmacy will deliver them to my house (Patient 1)

### The accessibility of NHS services

#### Limited access to health facilities (HCP appointments, follow-up)

Access to the NHS services is a top concern for patients with COPD. Patients reported the difficulty of seeing the medical staff at the surgery.

Well, you can't see a doctor. Yes, I have not seen my doctor for oh almost 2 years… And to get to speak, you have to explain everything to the receptionist before, and then you have to wait for a phone call from the doctor. Unless that is absolutely urgent that I need an appointment. And that’s only when I have my infection and I need an antibiotic… I can go to the pharmacist, which is where I live, where I have to go first. At the moment, I can manage to get to the pharmacy. (Patient 2)

The HCPs noted other concerns related to patients with COPD’ diagnosis, annual reviews and ability to check inhaler medication technique during the pandemic.

Massively. So we haven't done spirometry for kind of a year and a half or maybe a year and a quarter, a long time that we just stopped doing it…We've not been able to have official diagnosis with spirometry for a lot of patients with COPD (HCP 3)

Significantly, a lot of COPD reviews haven't happened during the pandemic last year. This means less face to face consultations and also with patients with COPD at increased risk of severe COVID so they are hesitant to come to GP surgery as well? And also, if they have exacerbation, it’s difficult to see at that stage of the initial consultation to have COVID or not so I think it’s affected a lot. (HCP 1)

#### Frustrations about the services provided by NHS

Three patients remarked on their frustration with the services provided by the NHS during the pandemic due to the lack of easy availability of doctors.

There’s no appointment, we can't come in, so that was it; we couldn't get in our doctor. So we had to go and see the doctor at the hospital (Patient 7)

I normally have or used to have breathing tests once every year at the surgery, you know, blowing something up into something to see what the pressure was inside. And I haven't had one now, for must be now two years or more (Patient 8)

#### Patients beliefs

37.5% of patients clearly stated that they avoid contacting the surgery during the pandemic due to their beliefs about the downside of productivity, and their reluctance to seek healthcare.

Well, I've been ill at a time, and I just haven't bothered asking to see a doctor because I know I can't see one (Patient 6)

Although the NHS services were viewed as busy during the pandemic, one patient with COPD living in the most deprived area stated that they understood the current situation and she had no objection to waiting longer for treatment.

I know that the National Health is in distress, so I don't push the doctor and I don't push the pharmacist; I will wait, I understand the problem that we deal with the national health at the moment (Patient 6)

### Health inequalities and e-health literacy

#### Patients’ digital disadvantage

Notably, many patients explained that using remote consultation technology—a video or phone consultation with healthcare professionals to discuss their health— was difficult for them, and they did not like it compared with the traditional method, which is a face-to-face consultation.

I'm not very familiar with the video call. I can assure you, I'm not that good with technology (Patient 6) 

I don't think it (remote consultation) would be detrimental, but I don't think it would particularly offer that much either. For me personally, probably no help at all (Patient 3)

Well, I haven't spoken to the doctor on the phone and I'd rather see the doctor face to face, and I haven't been able to do that. No, I don't want to get involved. That’s not right, you must see a doctor face to face (Patient 6)

In contrast, three patients with COPD said they welcomed the idea of using remote consultation for health safety purposes during the pandemic.

Well, yes, that would be nice. You know, it (remote consultation) would be safe at the moment (during the pandemic) (Patient 4)

To be quite honest, I sort of, I suppose I welcomed it (remote consultation) because it helped, and that’s the only reason I see (Patient 8)

HCPs raised some concerns regarding some patients using remote consultations, especially those related to communication difficulties, diagnosis and assessment of severity of respiratory illness.

I don't know if you've ever tried consulting with an elderly person on the phone, but it’s really hard like, communication difficulties, hearing difficulties (HCP 2)

I'd have to guess I think initially was a lot on the phone, not even seeing them, telling them on the phone, it sounds like COPD, try an inhaler, see how you get on for a month (HCP 3)

A GP who operated in a very deprived area highlighted the inequalities in access or ability to use the internet.

We have sent out like loads of COPD template patients, some of patients with COPD would not have replied because these patients who are not very good with computers, don't have Internet access. I work in a very deprived area. So the patient education is not very high (HCP 1)

HCPs positively perceived remote consultation as the main method during the pandemic. Some methods were used more frequently than pre-pandemic, such as telephone triage and the AccuRx template.

A lot of any information you can get beforehand through AccuRx. There are some templates already loaded up and if you send it to them, they are computer savvy; they will reply and get recorded to the patient notes. So you have a fair idea on the COPD control through that to decide because they give a CAT score whether they need to review them quickly or can it wait for a few more weeks, I think (HCP 1)

Interestingly, an HCP practising in the least deprived area discussed stretching time beyond usual during the remote consultation.

Everyone wants to see the GP in the flesh. Um. There’s no real reason why we need to see them face to face. That’s the honest truth. I can spend a bit more time compared to rushing through 10 minutes like it used to be; I can spend 15 minutes on the phone (HCP 3)

#### Inhaler technique

Clinicians reported remote checks of inhaler use were feasible but inferior to hands-on demonstration; many relied on signposting to reputable technique videos or on proxy metrics (eg, medication pickups), which can miss errors. Barriers were greatest for older or digitally excluded patients; enablers included video demonstrations and follow-up calls by respiratory nurses.

All HCPs stated concerns related to the demonstration of inhaler technique as they were unable to determine how well patients with COPD used their inhalers during remote consultations.

I think because it’s done remotely. I'm not sure how much we go into the detail about using their inhaler? (HCP 3)

Obviously, it’s been difficult in COVID because a lot of it had to be done over a video consultation. So you got an idea of how well they're using the inhalers, but, it’s still a bit of guesswork because you cannot; the patient is not there in front of you (HCP 2)

I don't think it’s a substitute. I think of face to face inhaler demonstration technique has a bigger impact. The only thing I can think of which I was asking my nurse also is to find out a video of inhaler techniques and then if we could upload them through AccuRx which we could send them to patients (HCP 1)

Inhaler technique might be an issue if they're 97 years old, or 98 years old, and they haven't got a smartphone and haven't got a link to the websites and the videos. It’s more of an issue. They should be shown really how to use the inhaler (HCP 3)

Notably, an HCP practising in the most deprived area discussed directing people more often to websites explaining how to use their inhalers correctly.

I have been doing a lot more is signposting patients to online resources about inhaler techniques. So I mean, mainly, to be honest, I've been using the Asthma UK website where they have a lot of seeing, a lot of the videos about inhalers, inhaler techniques, different inhalers, spacers, and either sending a link in a text message for the patient to look at it (HCP 4)

## Discussion

The current study aimed to explore the views of patients with COPD’ and HCPs on the impact of COVID-19 on the management of COPD, and specifically to explore remote consultation, its potential enablers and barriers. The findings highlighted three major themes: (a) coping and life adjustment, (b) the accessibility of NHS services and (c) health inequalities and e-health literacy. This study demonstrates that remote consultations were suitable for certain routine COPD requirements but were frequently less favoured than in-person care, especially among older adults and individuals with a higher disease burden, reflecting broader findings from telehealth assessments in COPD. Clinicians characterised remote care as feasible for stable patients but potentially uncertain for suspected exacerbations or diagnostic evaluation, consistent with reviews that advise against substituting physical examination in contexts of significant clinical uncertainty.^29^,^32^

### Findings in relation to existing literature

#### Patients’ difficulty to see medical staff and HCPs: suspension of lung function tests and reviews

Patients with COPD acknowledged the difficulty of accessing NHS services and HCPs, especially in seeking doctors’ support. Our finding is reinforced by other studies, which reported that accessibility to chronic care has decreased owing to the repositioning of medical professionals as a ‘call of duty’ for critical COVID-19 patients.^33^ Moreover, healthcare access has significantly decreased for chronic diseases such as COPD, hypertension and diabetes.^14^ HCPs in our study reported multiple concerns related to lung function tests and limited reviews. Lung function specialists examine patients with respiratory problems on a regular basis. However, during the pandemic, patients are required to take off their face masks during the examination, coughing when doing the manoeuvres, which increases the risk of viral transmission to healthcare staff, the testing environment and equipment. As a result, there was a substantial lack of access to pulmonary function testing services.^34^ Also affected has been the annual review check. NHS England and NHS Improvement have suggested that GPs could pause or suspend certain regular tasks to spare them time to cope with COVID-19. Officials addressed a letter to GPs and commissioners outlining a list of tasks that GPs should suspend or delay, including performing health checks on patients aged 75 and above and standard medical reviews ‘if in their view that is not the proper priority’.^35^

We identified service enablers such as pre-visit questionnaires (eg, CAT via AccuRx), triage and flexible appointment times, all consistent with digital pathways that collect symptom scores before clinician engagement. However, clinicians still expressed diagnostic caution for exacerbations and new presentations when objective examination or observation of work of breathing was indicated, and this was consistent with telemedicine guidance outlining the limits of remote respiratory assessment.^31 32 36^

#### Digital literacy and health inequalities

Our findings highlighted enablers and barriers of remote consultation. The remote consultation enablers include reduced exposure to pathogens and pollution (safety during the pandemic), some patients partially welcoming remote consultation, and remote consultation efficacy. On the contrary, several barriers were identified in our study, including difficulty around computer and technology use, patients’ age, the difficulty of communication, lack of non-verbal signs of HCPs, limited internet access, with some barriers exacerbated by deprivation. Many patients with COPD favoured a face-to-face consultation with their HCPs. Similarly, previous research identified an overwhelming preference for in-person consultations that continues to exist,^37 38^ and comparable results to ours in terms of the preferences of patients and their experiences with remote consultations.^39 40^

A key result of our study is the difficulties patients have interacting satisfactorily over the telephone. According to the Patient Information Forum, 1.7 million individuals in the UK struggle to adequately describe their illnesses or experiences over the phone, and 9 million individuals find it challenging to use digital technologies on their own.^12 13^ Prior research reported similar findings to ours, older persons, particularly those with limited health literacy, hearing or vision impairment or cognitive impairments, are more inclined to encounter obstacles related to health inequities.^13 41^ Deprivation is a significant barrier to providing remote consultation, associated with lower awareness and reduced inclination to use remote healthcare services.^13^

#### Inhaler technique: what works remotely

Clinicians often directed patients to device-specific instructional videos and were also generally more favourably inclined to video over telephone for technique coaching, consistent with evidence that video-based or virtual teaching to goal education improves inhaler skills and achieves results similar to in-person training.^42^ Reliance on remote proxies such as prescription refill or pickup rates may miss significant handling errors; therefore, remote education should, ideally, be complemented by targeted in-person reviews for digitally excluded patients, patients with continued and possibly incorrect techniques or unclear clinical status. Emerging adherence tools and electronic inhaler monitoring may supplement follow-up and reinforcement, but ideally as only adjuncts within a blended model that maintains access to hands-on assessment and coaching.^31 43^

Volpato *et al*^44^ demonstrated extensive telemedicine adoption in Italy, adaptation and discontinuity of care, which align with our study participants’ selective acceptance of remote consultations and their acknowledgement of the ongoing need for psychological and practical support. Pleguezuelos *et al*^45^ indicated elevated satisfaction levels for telephone consultations during the first phases of lockdown, despite the restructuring of consultations or missed appointments, implying that individuals may remain satisfied even when some in-person demands are unfulfilled. This challenge is crucial for service planning.

### Strengths and limitations

As far as we are aware, this is the first qualitative study concerning the views of both GPs and patients with COPD on the impact of COVID-19 on patients with COPD and in particular to explore potential enablers and barriers of using remote consultations. For the purpose of generalising our findings to patients in primary care in the West Midlands and other comparable places, the sample has good diversity in terms of education level and socioeconomic background/ level of deprivation, gender and symptom intensity, but less so for age group (as patients with COPD are generally older).

It was not feasible to conduct face-to-face interviews as planned because of the danger of contracting or transmitting COVID-19. Through warm, heartfelt, genuine greetings, active listening and a caring speech tone, interviews with patients helped them to feel relaxed and open up about difficult topics. Some patients with COPD shared confidential information about their diseases and other issues regarding their family members. However, the diversity of HCPs in the sample was not as broad as envisioned. This was due to the fact that no-one from other HCP groups agreed to take part in the study. We recognise that including only GPs likely tilted the themes towards primary care perspectives and under-represented pharmacy and nursing views. In addition, we acknowledge that not doing participant checking could affect credibility; however, we did conduct reflexive analysis, co-author verification of transcripts and the codebook, and provided a transparent audit trail. We additionally recognise that asking participants in 2021 to reflect on what had taken place a year before may have been harder for them to remember details. We acknowledge our research was at risk of selection bias as some groups would be more inclined to participate in the study than others. While an inductive approach to analysis allows for the development of themes from within the participants’ experiences, instantaneous analysis by a single researcher can introduce personal interpretation and potential bias.

### Practice and research implications

According to the recently released NHS Long Term Plan, technological advancements in primary healthcare will see a significant increase in usage of remote consultation.^46^ Some patients may embrace these changes; nevertheless, it is essential to recognise potential barriers such as deprivation and decline of some patients’ satisfaction towards the service. Further research is needed to thoroughly investigate the potential advantages and disadvantages of remote consultation, particularly video consultation, within the primary care context, including evaluating its cost-effectiveness.

In conclusion, patients with COPD and HCPs agreed that increased pressure on the health services during the pandemic led to a deterioration in providing COPD diagnosis and inhaler technique consultation. Considerable hurdles to overcome included those linked to health facilities access, patients’ beliefs towards remote consultations, and digital skills and difficulty of communication with an older cohort of patients, predominantly in deprived areas, for telemedicine services was highlighted to equip HCPs with the knowledge and abilities required to enhance care delivery. Lastly, there is no clear right or wrong way to conduct a remote consultation^37 38 47^; rather, there are benefits and drawbacks that can arise based on the specifics of the situation. Future lessons could be implemented post-pandemic, including rethinking how HCPs are trained to conduct remote consultations, emphasising building rapport and aspects related to therapy, providing flexibility in patient access to healthcare to ensure patients’ continuity of care and allocation of targeted and prioritised support to vulnerable patients with COPD to ensure their access to essential healthcare services, medication and supportive systems during periods of crisis.

## Supplementary material

10.1136/bmjresp-2025-003196 online supplemental file 1

## Data Availability

Data are available upon reasonable request.
