# Supplementary material for: COVID-19 and its impact on healthcare services provided to patients with COPD: a qualitative study
Source: BMJ Open Respir Res. 2025 Dec 25;12(1):e003196. doi: 10.1136/bmjresp-2025-003196 (PMC12742183; doi:10.1136/bmjresp-2025-003196)
Supplement: online supplemental file 1 [file bmjresp-12-1-s001.docx]

**Supplementary Data 1: The topic guides for patients with COPD and HCPs**

**Opening**

Thank you for taking part in this interview.

**HCP**

**Pandemic and remote consultation:**

[TDF: Beliefs about Capabilities]

How has the pandemic affected your ability to manage your patients with COPD?

How has the pandemic affected your ability to select/prescribe medications for patients with COPD especially inhalers?

How has the pandemic affected your ability to train/advise patients with COPD on how to take their inhaler medications?

Have remote consultations made a difference to this management?

[TDF: behavioural regulation]

1. Are there any aspects of the pandemic service changes which you plan to take forward in the future when managing your patients with COPD, especially when respiratory infections are widespread?

**Patients with COPD**

**Pandemic and remote consultation:**

[TDF: Beliefs about Capabilities]

How has the pandemic affected your consultations with the healthcare staff at the GP practice?

How has the pandemic affected your consultations/visits to the pharmacist?

[TDF: Emotion]

How do you feel about remote consultations?

Has having remote consultations/ the pandemic hindered your ability to look after your COPD and take your regular medications in any way?

What do you feel about learning how to use your inhalers using zoom for example? Or videos?

[TDF: Beliefs about Capabilities]

How do you think remote consultations by zoom or telephone would be useful in the future, especially when there are lots of infections going round?

**End of interview:**

**Conclusion questions**

• Do you have any other comments/remarks you would like to make on the topic?

Thank you for your contribution. I appreciate your time and thoughts.

**Supplementary Data 2: COREQ checklist**

Consolidated criteria for reporting qualitative studies (COREQ): 32-item checklist

Developed from:

Tong A, Sainsbury P, Craig J. Consolidated criteria for reporting qualitative research (COREQ): a 32-item checklist for interviews and focus groups. International Journal for Quality in Health Care. 2007. Volume 19, Number 6: pp. 349 – 357

| **Item No** | | **Guide Questions/Description** | **Reported on Page #** |  |
| --- | --- | --- | --- | --- |
| **Domain 1: Research team and reflexivity** | | | |  |
| **Personal Characteristics** | | | |  |
| 1. Interviewer/ facilitator | | Which author/s conducted the interview or focus group? | Pg 5 |  |
| 2. Credentials | | What were the researcher’s credentials? E.g., PhD, MD | Pg 5 |  |
| 3. Occupation | | What was their occupation at the time of the study? | Pg 5 |  |
| 4. Gender | | Was the researcher male or female? | Pg 5 |  |
| 5. Experience and training | | What experience or training did the researcher have? | Pg 5 |  |
| **Relationship with participants** | | | |  |
| 6. Relationship established | | Was a relationship established prior to study commencement? | Pg 5 |  |
| 7. Participant knowledge of the interviewer | | What did the participants know about the researcher? e.g. personal goals, reasons for doing the research? | Pg 5 |  |
| 8. Interviewer characteristics | | What characteristics were reported about the interviewer/facilitator? e.g. Bias, assumptions, reasons and interests in the research topic | Pg 6 |  |
| **Domain 2: study design** | | |  |  |
| **Theoretical framework** | | |  |  |
| 9. Methodological orientation and Theory | What methodological orientation was stated to underpin the study? e.g. grounded theory, discourse analysis, ethnography, phenomenology, content analysis | Pg 5 and 8 |  |  |
| **Participant selection** | | |  |  |
| 10. Sampling | How were participants selected? e.g., purposive, convenience, consecutive, snowball | Pg 5 |  |  |
| 11. Method of approach | How were participants approached? e.g., face-to-face, telephone, mail, email | Pg 5 |  |  |
| 12. Sample size | How many participants were in the study? | Pg 8 |  |  |
| 13. Non-participation Setting | How many people refused to participate or dropped out? Reasons? | N/A |  |  |
| 14. Setting of data collection | Where was the data collected? e.g., home, clinic, workplace | Pg 5 |  |  |
| 15. Presence of nonparticipants | Was anyone else present besides the participants and researchers? | N/A |  |  |
| 16. Description of sample | What are the important characteristics of the sample? e.g. demographic data, date | Pg 6 |  |  |
| **Data collection** | | |  | No |
| 17. Interview guide | Were questions, prompts, and guides provided by the authors? Was it pilot tested? | Pg 5 |  |  |
| 18. Repeat interviews | Were repeat interviews carried out? If yes, how many? | N/A |  |  |
| 19. Audio/visual recording | Did the research use audio or visual recording to collect the data? | Pg 8 |  |  |
| 20. Field notes | Were field notes made during and/or after the interview or focus group? | Pg 6 |  |  |
| 21. Duration | What was the duration of the interviews or focus group? | Pg 8 |  |  |
| 22. Data saturation | Was data saturation discussed? | Pg 5 |  |  |
| 23. Transcripts returned | Were transcripts returned to participants for comment and/or correction? | Pg 5 |  |  |
| **Domain 3: analysis and findings** | | |  |  |
| **Data analysis** | | |  |  |
| 24. Number of data coders | How many data coders coded the data? | Pg 8 |  |  |
| 25. Description of the coding tree | Did the authors provide a description of the coding tree? | Pg 8 |  |  |
| 26. Derivation of themes | Were themes identified in advance or derived from the data? | Pg 8 |  |  |
| 27. Software | What software, if applicable, was used to manage the data? | Pg 8 |  |  |
| 28. Participant checking | Did participants provide feedback on the findings? | Pg 5 |  |  |
| **Reporting** | | |  |  |
| 29. Quotations presented | Were participant quotations presented to illustrate the themes/findings? Was each quotation identified? e.g., participant number | Pg 9-14 |  |  |
| 30. Data and findings consistent | Was there consistency between the data presented and the findings? | Pg 9-14 |  |  |
| 31. Clarity of major themes | Were major themes clearly presented in the findings? | Pg 9-14 |  |  |
| 32. Clarity of minor themes | Is there a description of diverse cases or a discussion of minor themes? | Pg 9-14 |  |  |

**Supplementary Data 3: Summary of coding tree with themes, subthemes.**

**Coping and life adjustment**

- Changes to lifestyle: avoiding crowds; reduced socialising; altered shopping routines; modified work patterns; reduced exercise; prioritising home activities.​
- Impact of isolation/shielding/masking: feeling lonely; fear of exposure; fatigue from constant masking; missing family events; staying indoors; emotional strain.​
- Self‑management (carer dependency/roles): relying on caregiver for tasks; help with appointments; medication management support; learning self‑monitoring; difficulty with daily activities; increased caregiver burden.​

**Accessibility of NHS services**

- Limited access to facilities/follow‑up: cancelled clinics; long waits; phone‑only reviews; difficulty booking; fewer specialist slots; postponed routine care.​
- Frustrations about services: dissatisfaction with communication; feeling not listened to; unmet needs; fragmented care; limited continuity.​
- Beliefs about NHS capacity: system overwhelmed; staff shortages; resources stretched; reduced availability; concern about service sustainability.​

**Health inequalities and e‑health literacy**

- Patients’ digital disadvantage: no internet/device; low portal skills; trouble with video calls; navigation barriers; language/health‑literacy gaps; exclusion from virtual care.​
- Inhaler technique: incorrect steps; poor coordination; no spacer use; inadequate breath‑hold; device confusion; need for demonstration/review.​

**Consolidated categories (for the coding tree)**

- Coping and life adjustment consolidates the above codes into three subthemes with observed frequencies in patients: lifestyle changes 37.5%, isolation/shielding/masking 62.5%, and self‑management/caring roles 37.5%.​
- Accessibility of NHS services consolidates codes into limited access (75% patients; 100% HCPs), frustrations with services (37.5% patients), and beliefs about capacity (37.5% patients).​
- Health inequalities and e‑health literacy consolidate codes into patients’ digital disadvantage (100% patients and HCPs) and inhaler technique issues (100% HCPs).​
